# Holographic Microwave Image Classification Using a Convolutional Neural Network

**DOI:** 10.3390/mi13122049

**Published:** 2022-11-23

**Authors:** Lulu Wang

**Affiliations:** Biomedical Device Innovation Center, Shenzhen Technology University, Shenzhen 518118, China; wanglulu@sztu.edu.cn or lwang381@hotmail.com

**Keywords:** microwave imaging, breast cancer, deep learning, AlexNet, transfer learning

## Abstract

Holographic microwave imaging (HMI) has been proposed for early breast cancer diagnosis. Automatically classifying benign and malignant tumors in microwave images is challenging. Convolutional neural networks (CNN) have demonstrated excellent image classification and tumor detection performance. This study investigates the feasibility of using the CNN architecture to identify and classify HMI images. A modified AlexNet with transfer learning was investigated to automatically identify, classify, and quantify four and five different HMI breast images. Various pre-trained networks, including ResNet18, GoogLeNet, ResNet101, VGG19, ResNet50, DenseNet201, SqueezeNet, Inception v3, AlexNet, and Inception-ResNet-v2, were investigated to evaluate the proposed network. The proposed network achieved high classification accuracy using small training datasets (966 images) and fast training times.

## 1. Introduction

Breast cancer is the leading cause of female cancer deaths [1]. Previous studies showed that early breast cancer detection methods combined with suitable treatment could improve survival rates significantly [2]. X-ray mammography is the current gold-standard imaging tool for diagnosing breast cancer, but it produces harmful radiation and is unsuitable for dense breasts [3]. Microwave imaging has been proposed as one of the most potential breast imaging tools [4]. Researchers have extensively investigated microwave imaging in many aspects, including measurement of the microwave dielectric properties of breast tissues [5,6], image algorithms [7,8], numerical models [9,10], data acquisition systems [11,12,13], microwave antennas [14,15,16], clinical trials [17,18], image enhancement and improvement methods [19,20,21], and image classification [22,23,24]. If microwave images contain specific qualitative and quantitative indicators, this may help characterize benign and malignant tumors and predict disease. However, this work is challenging because this interdisciplinary study involves several disciplines, such as microwave science, medical imaging, machine learning, and computer vision. 

Over the past two decades, deep learning has attracted increasing attention and has achieved excellent performance in medical image classification and disease detection [25,26]. For example, Chen et al. employed the biclustering mining method in ultrasound images to identify breast lesions with accuracy, sensitivity, and specificity of 96.1, 96.7, and 95.7%, respectively [27]. However, the image datasets were too small to implement generalizations. Le et al. applied a deep neural network to enhance microwave images [28]. Khoshdel et al. investigated the feasibility of using 3D U-Net architecture to improve microwave breast images [29]. Rana et al. investigated machine learning for breast lesion detection using microwave radar imaging [22]. Mojabi et al. applied convolutional neural networks (CNN) to microwave and ultrasound images to classify uncertainty quantification and breast tissue [24]. However, obtaining big microwave image datasets for training networks is challenging. 

AlexNet is one of the most popular CNN architectures, and it is exploited on ImageNet datasets (including 50 million images) [30]. Previous studies demonstrated that small datasets (a few hundred) are employed for image classification [31]. However, small datasets are unsuitable for training networks due to easy overfitting. With the help of transfer learning, the training process can be conducted on a personal computer using small datasets [32]. 

In our previous studies, the holographic microwave imaging (HMI) method was proposed and tested for breast lesion detection [33,34,35]. This paper investigates the feasibility of using modified AlexNet with transfer learning to identify, classify, and quantify five classes of HMI datasets (fatty, dense, heterogeneously dense, very dense, and very dense breasts containing tumors), thereby solving the highly subjective judgment problem of lesions or abnormal tissues. Experimental validations are conducted on realistic MRI-based breast models to investigate the effectiveness and accuracy of modified AlexNet with transfer learning. In addition, a comparison study of several deep learning networks, including ResNet18, ResNet50, ResNet101, GoogLeNet, Inception v3, AlexNet, and VGG19, was conducted to evaluate the performance of HMI image classification. The research findings not only extend the application of deep learning but also help to understand microwave science from the perspective of deep learning with computer vision. The rest of this paper is organized as follows: Section 2 describes the proposed materials and method. Section 3 presents experimental validations and results. Section 4 concludes the study.

## 2. Materials and Method

### 2.1. Convolutional Neural Network 

A typical CNN contains an input layer (that receives pixel values), a convolution layer (that extracts image features), a pooling layer (that reduces the pixels to be processed and formulates abstract elements), and an output layer (that maps the extracted features into classification vectors corresponding to the feature categories) that can be described as:

(1) $$z^{l}=W^{l}*x^{l-1}+b^{l}\;a^{l}=\sigma \left(z^{l}\right)$$

where l denotes the lth layer and ∗ is a convolution operation. $W^{l}$, $b^{l}$, and $z^{l}$ denote the weights matrix, bias matrix, and weighted input of the lth layer. σ is the nonlinear activation function. When l=2, $x^{2-1}=x^{1}$ is the image matrix whose elements are pixel values. When l>2, $x^{l-1}$ is the feature maps matrix $a^{l-1}$, which is extracted from the (l−1)th layer, i.e., $x^{l-1}=a^{l-1}=\sigma \left(z^{l-1}\right)$. Let L be the output layer and $a^{L}$ is the final output vector. 

Nonlinear activation functions are employed from the second layer to the last layer. The cost function is:

(2) $$E_{0}^{L}=-\frac{1}{n}\overset{n}{\underset{i=1}{\sum }}\overset{N}{\underset{k=1}{\sum }}\left[t_{k}^{L}lna_{k}^{L}+\left(1-t_{k}^{L}\right)ln\left(1-a_{k}^{L}\right)\right]$$

where n is the training number and N is the number of neurons in the output layer corresponding to the N classes. $t_{k}^{L}$ is the targeted value corresponding to the kth neuron of the output layer and $a_{k}^{L}$ is the actual output value of the kth neuron of the output layer.

The output layer error can be defined as:

(3) $$\delta^{L}=\frac{\partial E_{0}^{L}}{\partial z^{L}}$$

where $\partial \left(\cdot \right)$ denotes the partial derivative operation. $l=\left\{\;L-1,\;L-2,\;\ldots ,\;2\right\}$, then:

(4) $$\delta^{l}=W^{l+1}\delta^{l+1}{}^{\circ}\sigma^{\prime }\left(z^{l}\right)$$

where ° is the Hadamard product. The partial derivative from $E_{0}^{L}$ to $W^{l+1}$ and $b^{l}$ can be calculated as follows:

(5) $$\left.\begin{array}{c} \frac{\partial E_{0}^{L}}{\partial W^{l}}=\frac{\partial E_{0}^{L}}{\partial a^{l}}{}^{\circ}\frac{\partial a^{l}}{\partial W^{l}}=\delta^{l}{}^{\circ}x^{l-1}\\ \frac{\partial E_{0}^{L}}{\partial b^{l}}=\frac{\partial E_{0}^{L}}{\partial a^{l}}{}^{\circ}\frac{\partial a^{l}}{\partial b^{l}}=\delta^{l} \end{array}\right\}$$

the changes can be computed by:

(6) $$\left.\begin{array}{c} ∆W^{l}=-\eta \frac{\partial E_{0}^{L}}{\partial W^{l}}\\ ∆b^{l}=-\eta \frac{\partial E_{0}^{L}}{\partial b^{l}} \end{array}\right\}$$

where η denotes the learning rate.

The ResNet architecture reduces training errors and network layers [36]. Adding a quick identity link to the primary network unit is the key to the ResNet architecture:

(7) $$H\left(X\right)=F\left(X\right)+X$$

where $H\left(X\right)$ is the ideal image and $F\left(X\right)$ is the residual map.

### 2.2. Datasets

**Figure 1:**
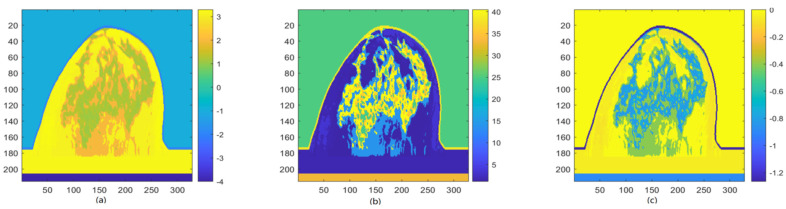
(**a**) example of 12 breast phantoms (breast 9); (**b**) real part of the relative complex-valued permittivity of breast 9; and (**c**) imaginary part of the relative complex-valued permittivity of breast 9.

**Figure 2:**
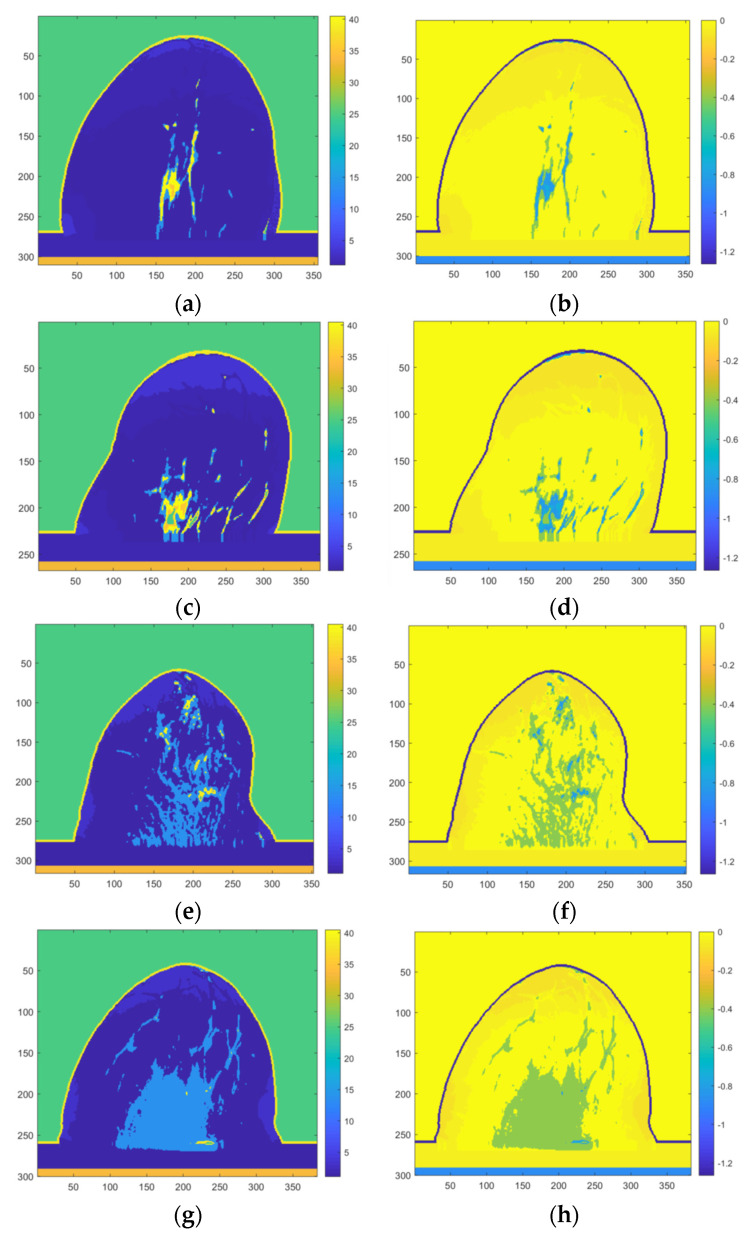

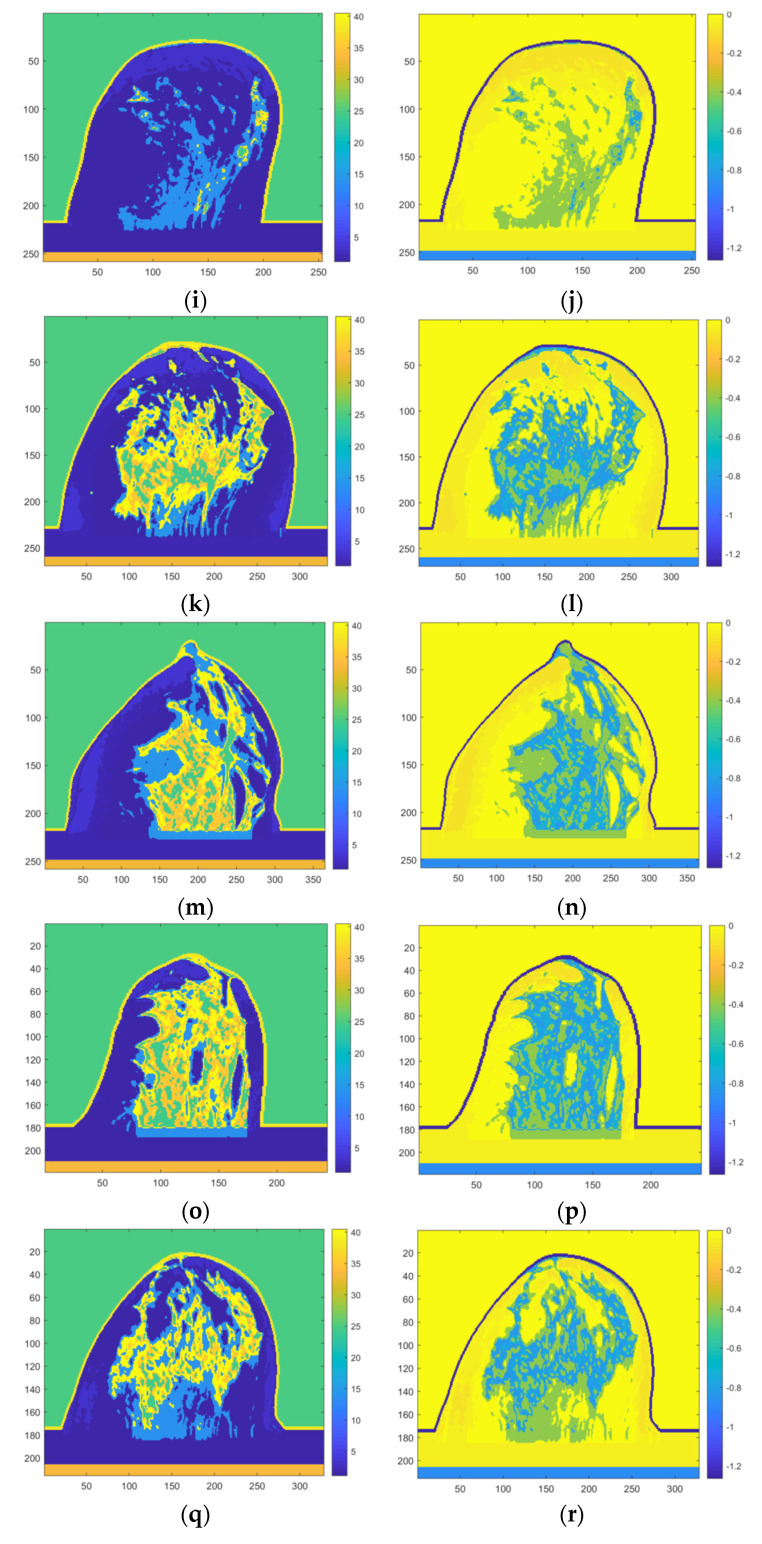

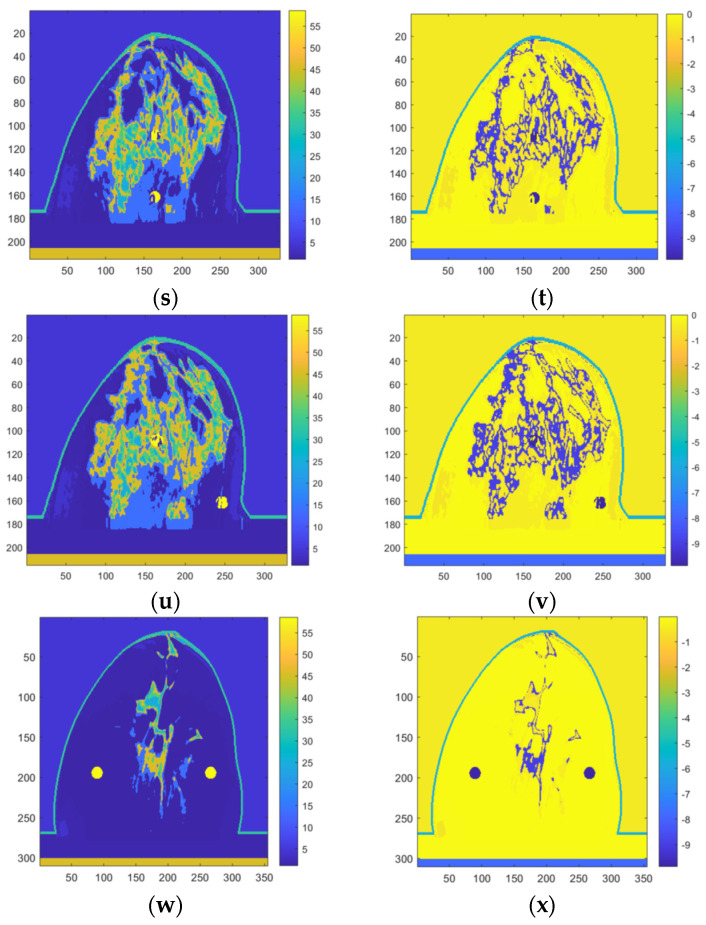
(**a**,**b**) real and imaginary parts of the relative complex-valued permittivity of breast 1; (**c**,**d**) real and imaginary parts of the relative complex-valued permittivity of breast 2; (**e**,**f**) real and imaginary parts of the relative complex-valued permittivity of breast 3; (**g**,**h**) real and imaginary parts of the relative complex-valued permittivity of breast 4; (**i**,**j**) real and imaginary parts of the relative complex-valued permittivity of breast 5; (**k**,**l**) real and imaginary parts of the relative complex-valued permittivity of breast 6; (**m**,**n**) real and imaginary parts of the relative complex-valued permittivity of breast 7; (**o**,**p**) real and imaginary parts of the relative complex-valued permittivity of breast 8; (**q**,**r**) real and imaginary parts of the relative complex-valued permittivity of breast 9; (**s**,**t**) real and imaginary parts of the relative complex-valued permittivity of breast 10; (**u**,**v**) real and imaginary parts of the relative complex-valued permittivity of breast 11 and; (**w**,**x**) real and imaginary parts of the relative complex-valued permittivity of breast 12.

As shown in Table 1, publicly available MRI-derived breast phantoms from 9 human subjects were used to develop realistic breast models by converting pixel values in MRI images to complex-valued permittivity [37,38]. Figure 1 shows a sample (breast 9) of 12 phantoms and the real and imaginary parts of the relative complex-valued permittivity. Figure 2 shows the real and imaginary parts of 12 breast phantoms. The HMI method was applied to generate HMI breast image datasets using the developed, realistic numerical microwave breast models. The numerical model simulated a sphere-shaped inclusion as a tumor (radius of 5 and 10 mm). 

This study used two datasets to train and test the CNN networks (see Table 2). Dataset 1 consists of the real part of HMI breast images, and dataset 2 consists of the imaginary part of HMI breast images. According to [37], the dataset in this study includes five classes of HMI images (12 phantoms), which are fatty, dense, heterogeneously dense, very dense, and breasts containing tumors. Class V was identified based on tumors that existed, and three Class V models were investigated in this study (see Table 1).

### 2.3. Training and Testing Data

#### 2.3.1. Image Segmentation 

An original HMI image contains different types of tissues with different sizes and cannot be applied directly for classification. We applied the image segmentation method to partition each original HMI image into sub-images and created the total of the sub-images. Sub-image properties are 227×227 pixels (a RGB image). The segmentation method helps to change the representation to a more meaningful and easier-to-analyze image while changing the scale to fit AlexNet. Image segmentation makes HMI images in each sub-image more uniform, which is suitable for classification and facilitates the final determination of the percentage of each mechanism. In addition, to ensure the authenticity of extracted features from the training dataset, image augmentation techniques such as rotation, height, and width shift were not used to ensure the integrity of the original images.

#### 2.3.2. Image Labeling

Both datasets 1 and 2 were classified into five classes (see Figure 2 and Table 2). The fatty breast (class I) consists of skin, muscle, and fat tissue. Dense breast (Class II) consists of skin, muscle, fat, and dense tissue (which has higher dielectric properties than fatty tissue). Heterogeneously dense breast tissue (Class III) consists of skin, muscle, fat tissue, and heterogeneously dense tissue. A very dense breast (Class IV) consists of skin, muscle, fat, dense tissue (which has higher dielectric properties than fat), and very dense fatty tissues (which have higher dielectric properties than fat and dense tissues). A breast contains tumors (Class V) consisting of skin, muscle, fat, heterogeneously dense tissue, and two tumors. 

The created HMI images illustrate the application behavior of the trained network. Therefore, their sub-images were not labeled. Different numbers of sub-images from each class were selected for manual labeling and then used for training and testing the proposed network. Training and testing datasets were utterly independent to ensure the reliability and stability of the proposed method.

For each dataset, 70% of the total images were used to train the proposed network, 20% of the total images were used to validate the network, and 10% of the total images were used to test the network. All breast image datasets were resized to 227 × 227 × 3 pixels. The training image dataset was applied to tune the network parameters using a gradient-based method. The testing image dataset was involved in the testing process to generate predictions. Table 2 shows the parameters used for training the networks. 

### 2.4. Network Architecture

#### 2.4.1. Modified AlexNet

AlexNet is the most popular CNN architecture due to its better performance in image classification. Thus, this study applied a modified AlexNet with transfer learning (see Table 3) to HMI images to improve image classification accuracy. Table 3 shows the structure of modified AlexNet with transfer learning. The first convolution layer of the network takes input datasets and passes them through convolution filters. Thus, the input image is required to be resized to 227 × 227 × 3 pixels, corresponding to the breadth, height, and three-color channels representing the depth of the input image. The last convolutional layer implements the reconstructed image process, aggregating the high-resolution patch-wise representations to produce the output image. The cross-entropy loss function is used to reduce errors. The batch normalization function is performed before each activation function to solve overfitting problems. The ReLU layer provides faster and more efficient training, mapping negatives, and maintaining positive values. The max pooling layer simplifies the output and reduces the resolution by reducing the number of parameters needed to learn. The fully connected layer combines all features to classify the images into four classes. The SoftMax function normalizes the output of the fully connected layer.

#### 2.4.2. Transfer Learning

As shown in Table 3, the last three layers of AlexNet were replaced by transfer learning to avoid overfitting. The proposed AlexNet network consists of a pre-trained network and a transferred network. The parameters in the pre-trained network were trained on publicly available ImageNet datasets. Therefore, it could be adapted to extract features from the HMI image dataset. The parameters in the transferred network represent a small part of the proposed AlexNet network. Thus, a small training dataset can meet the requirements of transfer learning. 

### 2.5. Data Analysis and Image Processing 

MATLAB version R2020a with the deep learning library tool was used for data analysis and image processing. The proposed network was developed on a laptop (ThinkPad P53) with an Intel i7-8700K CPU (2.60 GHz) and 256 GB of RAM. Stochastic gradient descent with momentum (SGDM) was selected to train the transferred part of AlexNet. 

The MATLAB Transfer Learning of Pretrained Network for Classification tool was used to train and test various deep learning networks using dataset 2, including ResNet18, GoogLeNet, ResNet101, VGG19, ResNet50, DenseNet201, SqueezeNet, Inception v3, AlexNet, and Inception-Res-Net-v2.

### 2.6. Performance Metrics

The overall performance of the proposed architecture depends on the evaluation matrix, which contains True Positives (TP), False Positives (FP), False Negatives (FN), and True Negatives (TN). The AlexNet architecture was evaluated on the testing dataset using four performance metrics, including precision and accuracy. Precision quantifies the exactness of a model and represents the ratio of carcinoma images accurately classified out of the union of predicted same-class images [39].

(8) $$\mathrm{Precision}\;=\frac{\mathrm{TP}}{\mathrm{TP}+\mathrm{FP}}$$

where TP refers to images correctly classified as breast tumor images and FP represents the typical images mistakenly classified as breast tumor images.

Accuracy evaluates the correctness of a model and is the ratio of the number of images accurately classified out of the total number of testing images.

(9) $$\mathrm{Accuracy}\;=\frac{\mathrm{TP}+\mathrm{FN}}{\mathrm{TP}+\mathrm{TN}+\mathrm{FP}+\mathrm{FN}}$$

where TN refers to the correctly classified standard images.

## 3. Results and Discussion

### 3.1. Results

**Figure 3:**
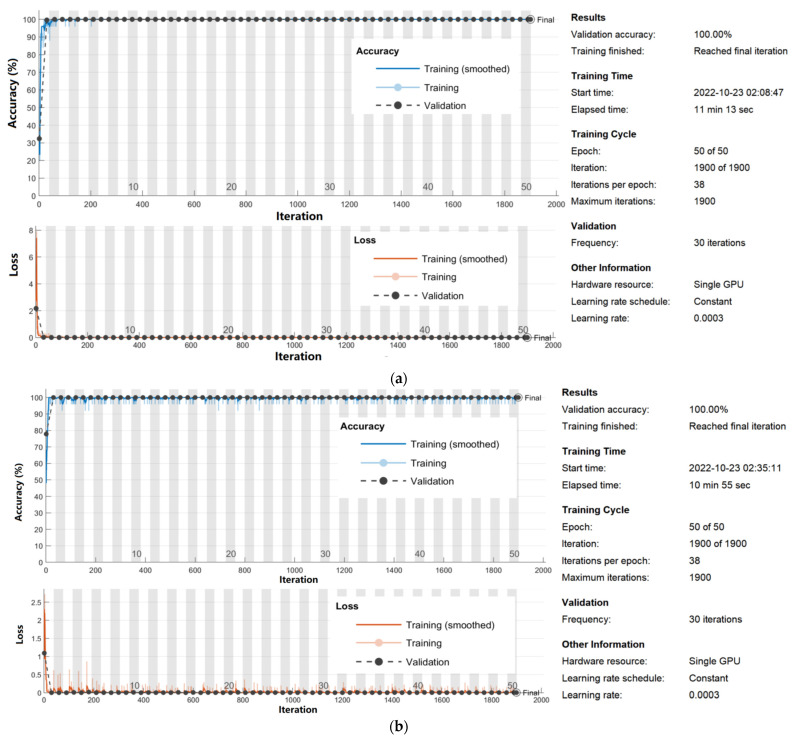
Training progress of the proposed network using (**a**) dataset 1 and (**b**) dataset 2.

Figure 3a shows the training progress of the proposed network using dataset 1 and the SGDM method, including classification accuracy and cross-entropy loss for each epoch of training and validation. At 50 epochs, the highest classification accuracy of training and validation was 100 and 100%, respectively, and the lowest cross-entropy loss of training and validation was 0 and 0%, respectively. The training time was 11 min and 13 s for training 966 images from dataset 1. 

Figure 3b displays the training progress of modified AlexNet with transfer learning using dataset 2 and the SGDM method. At 50 epochs, the highest classification accuracy of training and validation was 100 and 100%, respectively, and the lowest cross-entropy loss of training and validation was 0 and 0%, respectively. The training time was 10 min and 55 s for training 966 images from dataset 2. 

**Figure 4:**
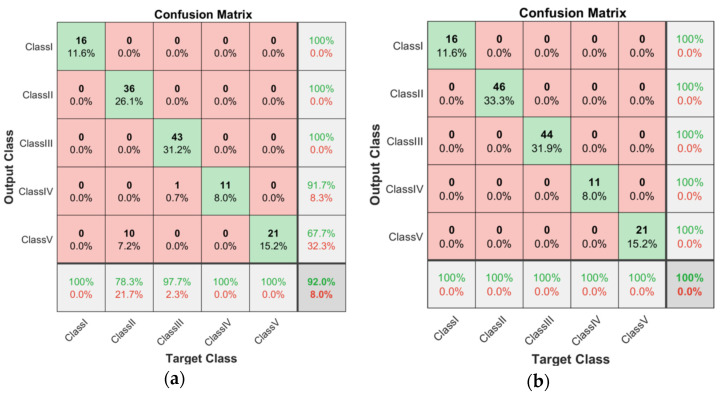
(**a**) Confusion matrix of testing dataset 1; (**b**) Confusion matrix of testing dataset 2.

As shown in Figure 4a, the performance of the proposed network was evaluated using the confusion matrix on testing images (from dataset 1). The actual horizontal row and predicted vertical column demonstrate the classification accuracy and sensitivity of the proposed network, respectively. For example, in the first row, 16 images were used to classify Class IV in the testing dataset, and 16 images (100%) were classified accurately. Therefore, the classification accuracy of Classes I, II, III, IV, and V was 100, 100, 100, 91.7, and 67.7%, respectively. In the first column, 16 images were used to predict class I of the testing images (from dataset 1), where 16 images (100%) were classified accurately. The sensitivity of Classes I, II, III, IV, and V was 100, 78.3, 97.7, 100, and 100%, respectively. 

Figure 4b shows the performance of modified AlexNet with transfer learning on testing images (from dataset 2). In the first row, 16 images were used to classify Class I in the testing dataset, and 16 images (100%) were classified accurately. The proposed network obtained a classification accuracy of 100, 100, 100, 100, and 100% for Classes I, II, III, IV, and V, respectively. In the first column, 16 images were used to predict Class I in the testing images, where 16 images (100%) were classified accurately. The proposed network obtained a sensitivity of 100, 100, 100, 100, and 100% for Classes I, II, III, IV, and V, respectively.

**Figure 5:**
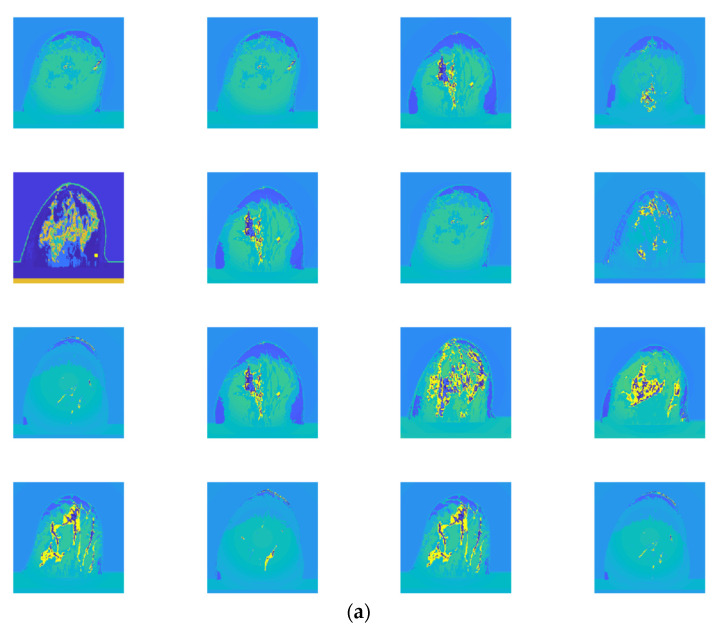

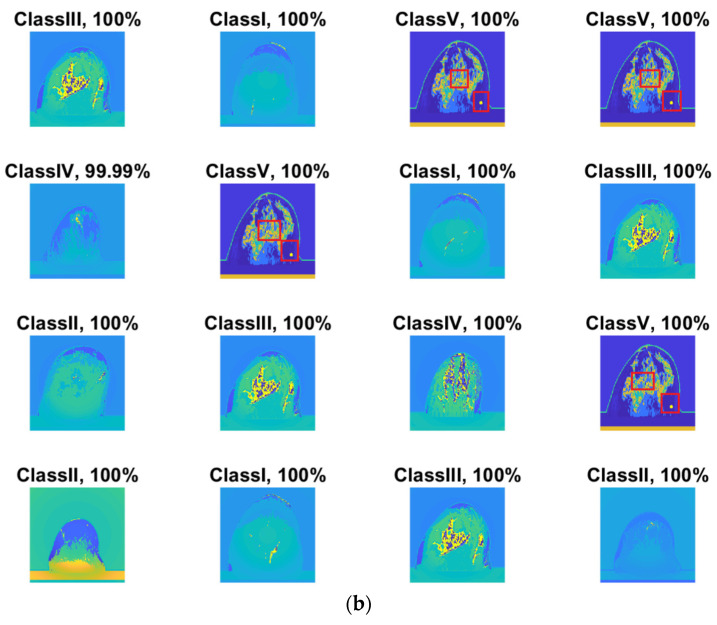
(**a**) randomly selected 16 examples of training images (from dataset 1), and (**b**) randomly selected 16 examples of testing images (from dataset 1).

Figure 5a,b demonstrate the randomly selected 16 examples of training images (from dataset 1) and randomly selected 16 examples of testing images (from dataset 1) using AlexNet with a transfer learning network, respectively. 

**Figure 6:**
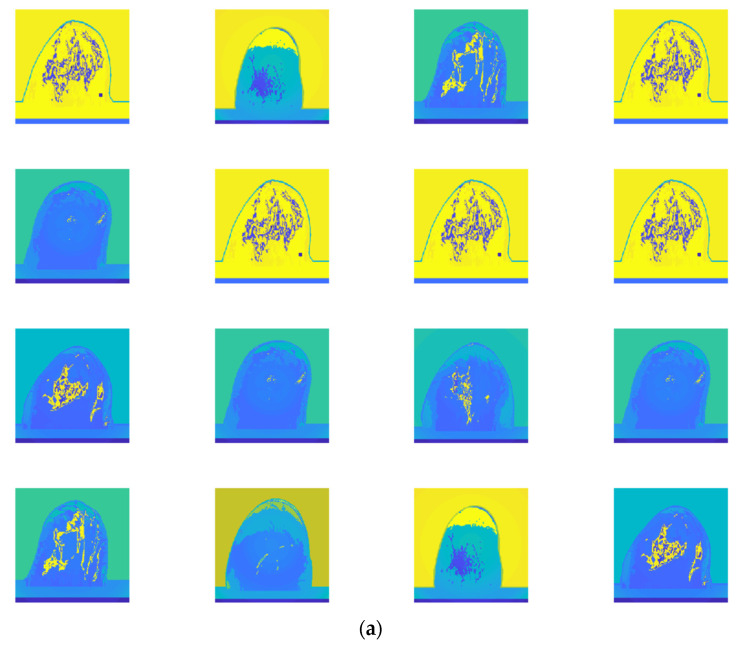

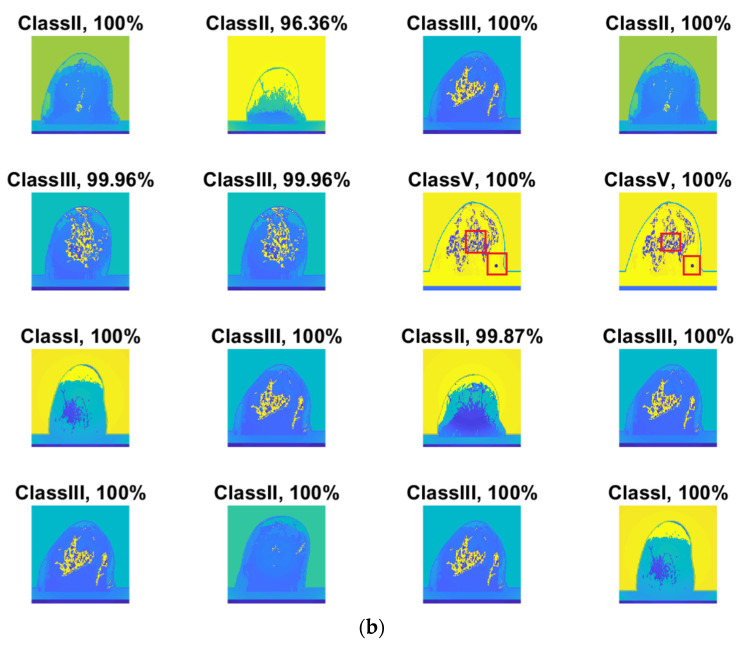
(**a**) randomly selected 16 examples of training images (from dataset 2); (**b**) randomly selected 16 examples of testing images (from dataset 2).

Figure 6a,b display the randomly selected 16 examples of training images (from dataset 2) and randomly selected 16 examples of testing images (from dataset 2), respectively. 

Table 4 presents the prediction results of dataset 2 using several deep learning networks. MobileNet-v2 obtained the highest accuracy (96.84%), and the training time was 28 min and 38 s. AlexNet used the shortest training time (3 min and 4 s) with relatively low accuracy (79.89%), Inception-ResNet-v2 obtained the lowest accuracy (79.34%) and used a long training time (106 min and 48 s), and DenseNet201 used the longest training time (132 min and 25 s) with relatively high accuracy (96.01%). Modified AlexNet with transfer learning achieved higher classification accuracy than other deep learning networks, which is suitable for classifying HMI images.

### 3.2. Discussion 

In this study, five classes of breast phantoms were developed using the method presented in [37]. The initial HMI breast images were created using the HMI method detailed in [33]. The initial images were analyzed and processed using the proposed CNN architecture. The proposed architecture offered higher classification accuracy and sensitivity for image dataset 2 (imagery-part HMI images; see Figure 4a) than image dataset 1 (real-part HMI images; see Figure 4b). For image dataset 1, the modified AlexNet with transfer learning offers higher classification accuracy for classes I–III (100%) than classes IV (91.7%) and V (67.7%), and higher sensitivity for classes I (100%), IV (100%), and V (100%) than classes II (78.3%) and III (97.7%). However, no significant difference in classification accuracy and sensitivity was obtained for dataset 2. Figure 4 demonstrates that image datasets affect the performance classification accuracy and sensitivity of the modified AlexNet with transfer learning.

The randomly selected 16 testing examples of image dataset 1 are shown in Figure 5b, and the 16 randomly selected testing examples of image dataset 2 are shown in Figure 6b. Although a classification accuracy of 100% was obtained for examples of image dataset 1 (see Figure 5b), it does not mean that the classification accuracy of dataset 1 is as high as 100%. For example, the classification accuracy rates of 91.7% and 67.7% were obtained for classes IV and V, respectively (see Figure 4a). Although the proposed CNN architecture provides accuracy and sensitivity of 100% to classify dataset 2 (see Figure 4b), the classification accuracy of some testing examples is below 100% (96.36–99.96%; see Figure 6b). This may be caused by MATLAB calculation errors. 

Compared with some popular deep learning networks (see Table 4), modified AlexNet with transfer learning has apparent advantages in classification accuracy and training time. For example, modified AlexNet with transfer learning obtained higher accuracy (100% vs. 96.84%) and required shorter training time (10 min 55 s vs. 28 min 38 s) to classify image dataset 2 than MobileNet-v2. The experimental results demonstrated that the modified AlexNet with transfer learning could identify, classify, and quantify HMI images with high accuracy, sensitivity, and reasonable training time. Several factors may affect the test results, including image preprocessing, the number of training images (in percentages), the total number of image datasets, and MATLAB calculation errors.

## 4. Conclusions

In this study, the CNN architecture was introduced for analyzing HMI images. A modified AlexNet with transfer learning was developed to identify, classify, and quantify five classes of HMI images (fatty, dense, heterogeneously dense, very dense, and very dense breasts containing tumors). Various experimental validations were conducted to validate the performance of the proposed network. Various popular deep learning networks, including AlexNet, were studied to evaluate the proposed network. Results demonstrated that the proposed network could automatically identify and classify HMI images more accurately (100%) than other deep learning networks. In conclusion, the proposed network has the potential to become an effective tool for analyzing HMI images using small training datasets, which offers promising applications in the microwave breast imaging field.

## Figures and Tables

**Table 1:** Characteristics of breast phantoms.

Number	Phantom Class	Quantity	Model	Size
No 1	I: fatty	253	RGB	310×355×253
No 2	I: fatty	288	RGB	267×375×288
No 3	II: dense	307	RGB	316×352×307
No 4	II: dense	270	RGB	300×382×270
No 5	II: dense	251	RGB	258×253×251
No 6	III: heterogeneously dense	202	RGB	269×332×202
No 7	III: heterogeneously dense	248	RGB	258×365×248
No 8	III: heterogeneously dense	273	RGB	219×243×273
No 9	IV: very dense	212	RGB	215×328×212
No 10	V: very dense breast contains two tumors	212	RGB	215×328×212
No 11	V: very dense breast contains two tumors	212	RGB	215×328×212
No 12	V: fatty breast contains two tumors	253	RGB	310×355×253

**Table 2:** Training parameters.

Dataset	1	2
Modality	Real part of HMI breast	Imaginary part of HMI breast
Number of phantoms	12	12
Classes of images	5	5
Number of HMI images	1379	1379
Image size	227 × 227 × 3	227 × 227 × 3
Number of training images	966	966
Number of validation images	275	275
Number of test images	138	138
Number of Class I	160	160
Number of Class II	457	457
Number of Class III	444	444
Number of Class IV	108	108
Number of Class V	210	210
Cross-validation group	8-fold	8-fold
Maximum number of epochs	50	50
Minimum batch size	25	25
Validation frequency	30	30
Initial learning rate	0.0003	0.0003

**Table 3:** AlexNet with transfer learning.

Schematic	No.	Name	Type	Activations	Weights & Bias
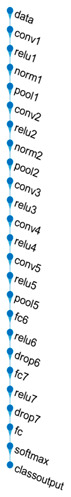	1	data	Image input	227 × 227 × 3	
	2	conv1	Convolution	55 × 55 × 96	Weights: 11 × 11 × 3 × 96; bias: 1 × 1 × 96
	3	relu1	ReLu	55 × 55 × 96	
	4	norm1	Cross-channel normalization	55 × 55 × 96	
	5	pool1	Max pooling	27 × 27 × 96	
	6	conv2	Grouped convolution	27 × 27 × 96	
	7	relu2	ReLU	27 × 27 × 256	Weights: 5 × 5 × 48 × 128; bias: 1 × 1 × 128 × 2
	8	norm2	Cross-channel normalization	27 × 27 × 256	
	9	pool2	Max pooling	13 × 13 × 256	
	10	conv3	Convolution	13 × 13 × 384	Weights: 3 × 3 × 25 × 384; bias: 1 × 1 × 384
	11	relu3	ReLU	13 × 13 × 384	
	12	conv4	Grouped convolution	13 × 13 × 384	Weights: 3 × 3 × 192 × 192; bias: 1 × 1 × 192 × 2
	13	relu4	ReLU	13 × 13 × 384	
	14	conv5	Grouped convolution	13 × 13 × 256	Weights: 3 × 3 × 192 × 128; bias: 1 × 1 × 128 × 2
	15	relu5	ReLU	13 × 13 × 256	
	16	pool5	Max pooling	6 × 6 × 256	
	17	fc6	Fully connected	1 × 1 × 4096	Weights: 7029 × 9216; bias: 4096 × 1
	18	relu6	ReLU	1 × 1 × 4096	
	19	drop6	Dropout	1 × 1 × 4096	
	20	fc7	Fully connected	1 × 1 × 4096	Weights: 4096 × 4096; bias: 4096 × 1
	21	relu7	ReLU	1 × 1 × 4096	
	22	drop7	Dropout	1 × 1 × 4096	
	23	fc8	Fully connected	1 × 1 × 4	Weights: 4 × 4096; bias: 4 × 1
	24	softmax	SoftMax	1 × 1 × 4	
	25	output	Classification output		

**Table 4:** HMI image classification using different deep learning networks.

Architecture	Accuracy	Training Time	Result
MobileNet-v2	96.84%	28 min 38 s	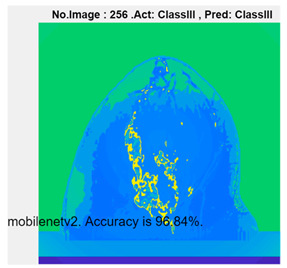
DenseNet201	96.01%	132 min 25 s	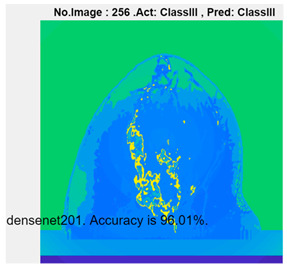
SqueezeNet	92.98%	16 min 3 s	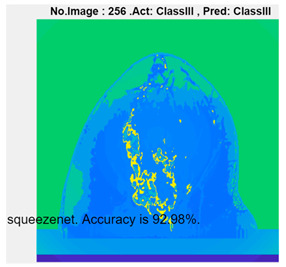
Inception-v3	86.24%	11 mins 30 s	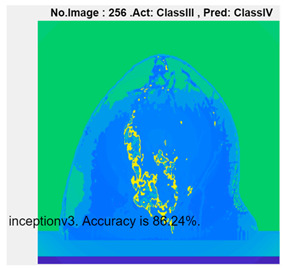
ResNet101	84.73.%	43 min 5 s	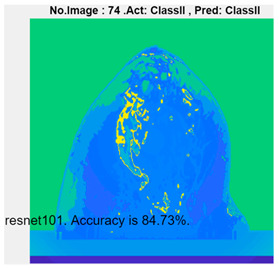
GoogLeNet	81.02%	7 min 48 s	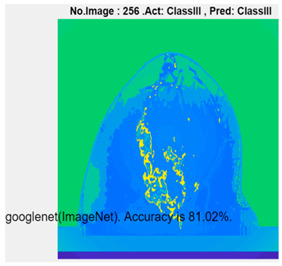
AlexNet	80.33%	5 min 39 s	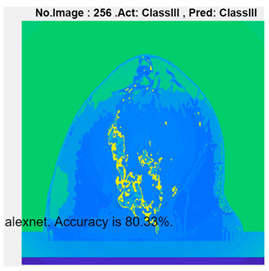
ResNet50	78.40%	36 min 16 s	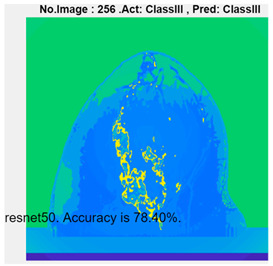
ResNet18	77.30%	11 min 45 s	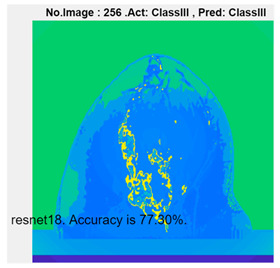
Inception-ResNet-v2	73.18%	106 mins 48 s	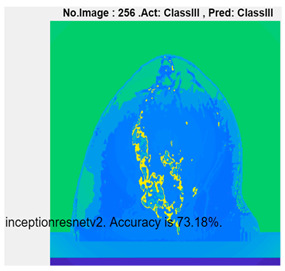

## Data Availability

Data and code are available from the corresponding authors upon reasonable request.
